# Spatial Distributions of Diarrheal Cases in Relation to Housing Conditions in Informal Settlements: A Cross-Sectional Study in Abidjan, Côte d’Ivoire

**DOI:** 10.1007/s11524-023-00786-z

**Published:** 2023-10-06

**Authors:** Vitor Pessoa Colombo, Jérôme Chenal, Brama Koné, Jeanne d’Arc Koffi, Jürg Utzinger

**Affiliations:** 1 École Polytechnique Fédérale de Lausanne, Lausanne, Switzerland; 2 École Polytechnique Fédérale de Lausanne, Lausanne, Switzerland; 3 Université Mohammed VI Polytechnique, Ben Guerir, Morocco; 4 Centre Suisse de Recherches Scientifiques en Côte d’Ivoire, Abidjan, Côte d’Ivoire; 5 Université Péléforo Gon Coulibaly, Korhogo, Côte d’Ivoire; 6 Centre Suisse de Recherches Scientifiques en Côte d’Ivoire, Abidjan, Côte d’Ivoire; 7 Swiss Tropical and Public Health Institute, Allschwil, Switzerland; 8 University of Basel, Basel, Switzerland

**Keywords:** Abidjan, Diarrhea, Informal settlements, Join count statistic, Precarious housing, Spatial clusters, Water, sanitation, and hygiene, WASH

## Abstract

In addition to individual practices and access to water, sanitation, and hygiene (WASH) facilities, housing conditions may also be associated with the risk of diarrhea. Our study embraced a broad approach to health determinants by looking at housing deprivation characteristics as exposures of interest and confronting the latter’s spatial distribution to that of diarrheal cases. We tested the hypothesis that the risk of diarrhea in informal settlements is not only associated with WASH services, but also with inadequate dwelling characteristics, and that their spatial distributions follow similar patterns. We designed a cross-sectional study and collected primary data through georeferenced household surveys in two informal settlements in Abidjan, Côte d’Ivoire. We used local join count statistics to assess the spatial distribution of events and multiple logistic regressions to calculate adjusted odds ratios between diarrhea and exposures. A total of 567 households were enrolled. We found that constant access to basic WASH services, non-durable building materials, cooking outdoors, and water service discontinuity were associated with higher risks of diarrhea in the general population. The spatial distribution of diarrheal cases coincided with that of dwelling deprivation characteristics. We observed significant heterogeneity within the study sites regarding the spatial distribution of diarrheal cases and deprived dwellings. Along with WASH infrastructure, communities also need dignified housing to effectively prevent diarrhea. We recommend that decision-makers acknowledge a “spectrum” of deprivation within the heterogeneous universe of informal settlements, adopting a site-specific approach based on high-resolution data to address diarrhea and improve people’s well-being.

## Introduction

Diarrheal diseases remain a global health challenge, as they are among the top five causes of disability-adjusted life years (DALYs) according to the 2019 Global Burden of Disease Study [1]. This burden disproportionally affects children under the age of 5 in sub-Saharan Africa (SSA). In 2016, out of the 446,000 estimated deaths in this age group caused by diarrhea, 290,724 (65%) occurred in SSA [2]. The latter is also one of the fastest-urbanizing regions in the world [3]. This brings along considerable challenges for the provision of water, sanitation, and hygiene (WASH) services and, consequently, the prevention of numerous illnesses, notably diarrhea [4].

One of the main challenges faced by cities in SSA is the occurrence of “informal” urbanization, characterized by extralegal land occupation, and the dissociation between urban growth and the extension of basic infrastructure. In 2020, more than 230 million urbanites (50.2% of the urban population in SSA) lived in “slum” households [5]. According to the United Nations Human Settlement Programme (UN-Habitat), these households experience one or several of the following deprivations: (i) lack of access to improved water and sanitation facilities, (ii) overcrowded and precarious housing conditions and location, (iii) voicelessness and powerlessness in political systems and governance processes, and (iv) lack of tenure security [5]. Here, we opted to refer to settlements facing any of the deprivations above as “informal settlements,” given earlier debates on the pejorative nature of the term “slum” [6].

The risk of diarrheal diseases is closely related to access to WASH amenities, which are essential to interrupt contamination pathways, particularly the fecal-oral route [7], but are often lacking in informal settlements [4, 5]. The international literature has extensively assessed the relationship between diarrhea and WASH services [2, 7, 8]. Additionally, previous studies have focused on factors other than WASH that might govern contamination pathways, notably poor housing conditions and cohabitation with animals [9], food handling [10], and exposure to environmental sources of pathogens in public spaces [11]. These studies reflect a renewed interest in environmental health determinants and raise the need for a broader approach to address variations in morbidity and mortality patterns across populations based on socioeconomic and housing conditions [12]. These studies also draw attention to the complexity of causal pathways for diarrhea by demonstrating how, beyond individual hygiene practices and access to WASH facilities, community-level and dwelling conditions are relevant exposures.

Our study is inscribed within this broader approach by looking at specific housing deprivation characteristics as environmental exposures of interest and confronting the latter’s spatial distribution to that of diarrheal cases (health outcomes of interest). Although previous studies have already assessed relations between housing deprivation and diarrheal diseases in SSA [9, 13], besides a few exceptions [14, 15], there is a paucity of empirical evidence elaborated on a cartographic basis and at a high spatial resolution (i.e., with precise maps of the built environment, housing characteristics, and cases of diarrhea). This “spatially explicit” approach is critical for urban planners to assess disease risk factors related to the built environment and plan tailored interventions. Such an approach is particularly relevant in informal settlements, where “neighborhood effects” caused by inadequate living conditions exacerbate the risk of numerous illnesses, including diarrhea [4]. Moreover, considering the differences that exist between and within informal settlements [16], spatial analyses based on high-resolution health data can provide invaluable insights to better understand environmental health determinants in those vulnerable settings.

Building on our recent research [17, 18] and on previous environmental health studies in SSA [9, 13], this study argues for a holistic approach to the prevention of diarrheal diseases that, beyond WASH services, also addresses the physical environment and the spatial characteristics of poverty [19]. Our starting point was the assumption that inadequate dwelling conditions hamper the potential health benefits expected from WASH interventions, which can be indirectly measured by the risk of diarrhea [20]. We tested the hypothesis of whether, in addition to access to adequate WASH services, the risk of diarrhea in informal settlements is associated with dwelling deprivation features.

## Materials and Methods

### Study Design and Geographic Scope

We designed a cross-sectional study. Primary data were collected through household surveys in two informal settlements in Abidjan, Côte d’Ivoire. Given its rapid spatial and demographic growth and its “urban primacy” [3], Abidjan is a city that well illustrates urbanization processes in SSA. Like other cities in the region, it faces challenges related to access to basic services, notably WASH [21], and a significant share of the population living in informal settlements [22, 23].

**Fig. 1:**
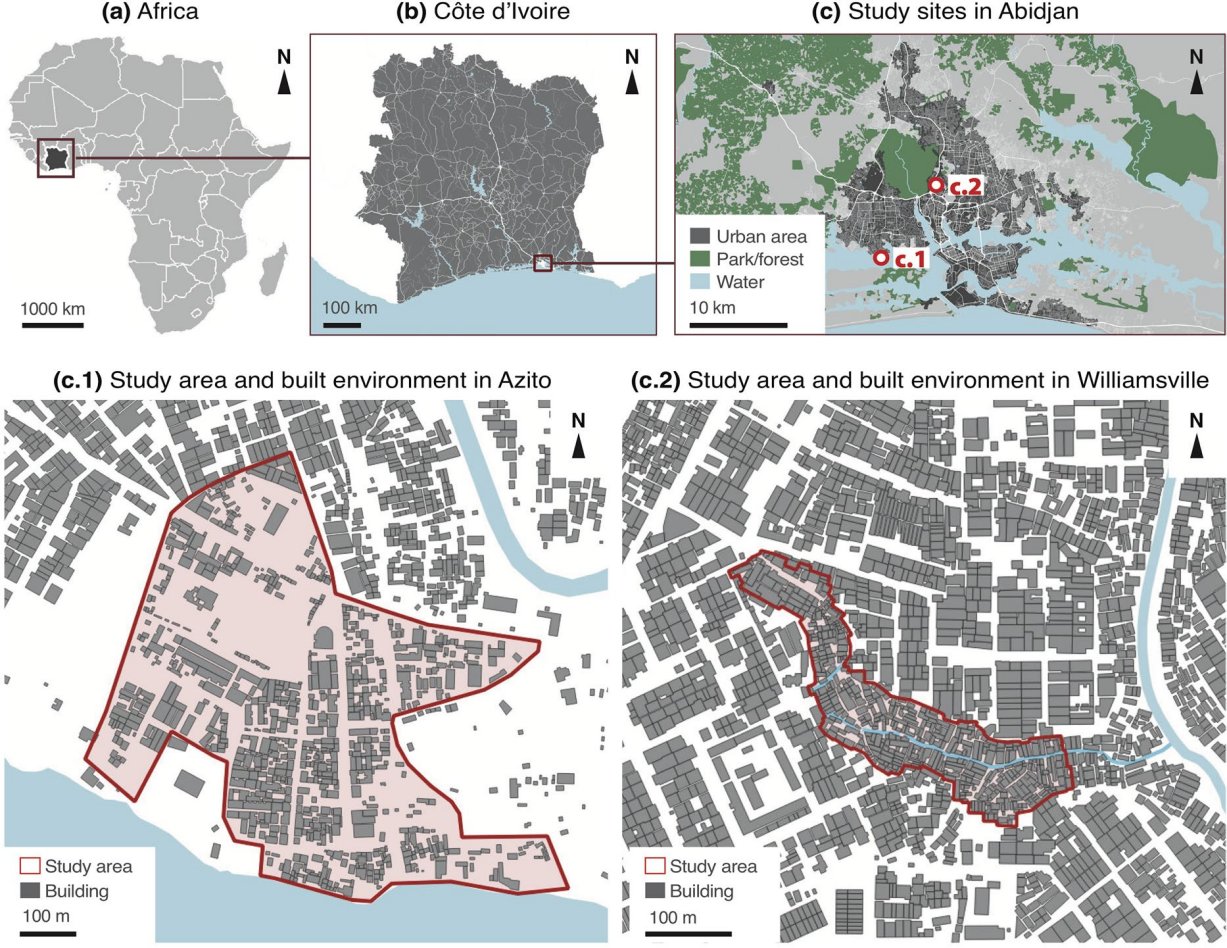
Geographic location of the study sites in Abidjan, Côte d’Ivoire. Elaborated from GADM, OpenStreetMap, Ecopia Building Footprints © 2021 Ecopia Tech Corporation, Imagery © 2021 DigitalGlobe, Inc

We purposefully selected two settlements, Azito and Williamsville (Fig. 1). This selection was motivated by ensuring sufficient variations in the exposure variables of interest, i.e., housing characteristics. Indeed, the two sites differ in their social and spatial compositions. Azito is an old fishermen’s village traditionally occupied by the Ébrié people, located in a peripheral area of Abidjan by the Ébrié Lagoon. Although it is considered an informal settlement for the lack of tenure security and sanitation infrastructure, it is less densely built than most informal settlements, with large open spaces. Its population is relatively wealthy and well-educated. Conversely, Williamsville is very densely built and situated in a central area near referential commercial locations. Its population is heterogenous (with diverse geographic origins) and economically vulnerable compared to other sites in Abidjan. In addition to poverty, people in Williamsville face a lack of tenure security, a severe lack of sanitation services (being exposed to open sewerage and garbage disposal), and seasonal flooding events. Accessibility to the sites and acceptance of the study by residents (represented by several “village chiefs”) were additional features guiding our selection.

### Outcome of Interest and Sample Size

The outcome used to determine the sample size was diarrhea, i.e., the passage of three or more loose or liquid stools per day [24]. Following a previous diarrhea study in Abidjan [25], we detected cases of diarrhea occurring in the 2 weeks preceding the survey. Because the burden of diarrhea is higher among children below the age of 5 [2], we used the estimated prevalence in this age group as a parameter for the sample size calculation. The latter is given by Eq. (1), based on existing guidelines for prevalence studies [26]:

1 $${\boldsymbol{n}}_{\textbf{0}}=\frac{{{\boldsymbol{Z}}^{\textbf{2}}}_{\textbf{1}-\propto /\textbf{2}}\times \boldsymbol{P}\times \left(\textbf{1}-\boldsymbol{P}\right)\times {\boldsymbol{D}}_{\boldsymbol{e}\boldsymbol{ff}}}{{\boldsymbol{e}}^{\textbf{2}}}$$

where ***n***_**0**_ is the minimum number of individuals, ***Z***_**1** −  ∝ /**2**_ is the critical value for the standard normal distribution corresponding to a type I error of **α** (here, α = 0.05; thus, ***Z***_**1** −∝ /**2**_ = 1.96), ***P*** is the expected diarrhea prevalence given by prior studies [25] (here, ***P*** = 0.15), ***D***_***eff***_ is the “design effect” (here, ***D***_***eff***_ = 1.5), and ***e*** is the margin of error at the 95% confidence level (***e*** = 0.05). Because the survey’s unit was the household—not the individual—***n***_**0**_ was adjusted to correspond to the minimum number of households. It was adjusted by: (i) the proportion of the targeted population (children younger than 5 years, i.e., 15%), (ii) average household size (4.5 individuals), and (iii) the expected valid response rate (here, 90%), considering potential data entry errors. These calculation parameters resulted in a minimum of 484 households.

### Exposures of Interest

We selected several housing deprivation features as exposures of interest. To define these features, we considered the housing components emphasized by UN-Habitat in the definition of “slum” [5], i.e., construction materials and overcrowding. If the walls, roof, or floor was made of non-durable materials (e.g., rudimentary planks or unfinished surfaces), the dwelling was considered “inadequate,” and if the household had more than three individuals per room, it was considered “overcrowded.” In addition, as foodborne infections are the main cause of diarrhea [10], we observed whether the household had an indoor cooking space, assuming this may protect food from external contamination sources such as flies or dirt.

We also considered access to WASH services, which were categorized as “at least basic” or “not basic,” adhering to definitions put forth by the World Health Organization (WHO) and the United Nations Children’s Fund (UNICEF) in their Joint Monitoring Programme for Water, Sanitation, and Hygiene (JMP) [27]. As recommended by the JMP [27], we included a question on whether the household had constant access to water in the month preceding the survey. Finally, we considered the educational attainment of the household head and constructed an asset-based wealth index as control variables for socioeconomic status, as they may lead to a recall bias affecting the count of self-reported diarrheal cases [28].

### Data Collection and Inclusion Criteria

The household surveys were conducted in February 2022, thus avoiding the rainy season, which could impose accessibility challenges and bias our findings by affecting the incidence of diarrhea. Before the surveys, 10 data collectors followed a 4-day training to be familiarized with the household questionnaire and the tools used, i.e., the mobile application KoboCollect 1.30.1 (Kobo; Cambridge, USA) operated on tablets (Galaxy Tab A 8.0 2019, by Samsung; Suwon-si, South Korea).

The investigators and the village chiefs collaboratively defined the study sites’ perimeters (Fig. 1). Each site was divided into five areas having an equivalent number of built structures (remotely detected from satellite imagery), which served to estimate the number of households in each area. Data collectors were organized into five groups of two and were instructed to select one out of two addresses, following a random walk trajectory [29] within their respective areas. All surveys were geo-referenced to the household’s location. Only adults who had resided for 2 or more weeks within the study sites’ perimeters were eligible to answer the surveys.

### Statistical Analysis

The analyses were done in Python language (see Supplementary Materials). We used multiple logistic regressions (MLRs) to test associations between diarrhea and exposure variables derived from dwelling conditions, controlling for access to WASH services and socioeconomic characteristics. Associations with the risk of diarrhea were inferred through adjusted odds ratios (aORs) obtained from the MLRs and the 95% confidence interval (CI). An association was considered significant if the aOR’s 95% CI excluded 1 and the logistic model’s overall fit was acceptable—i.e., a likelihood ratio test (LLR) *P* value <0.05. The MLRs were stratified; the first model included all participants (general population), while the second included only children younger than 5 years.

To build the MLRs (Table 1), we first ran a preliminary selection of explanatory variables through bivariate logistic regressions, using diarrhea as the dependent variable. A variable was pre-selected if its beta parameter’s *P* value was below 0.1. Then, we calculated the pre-selected variables’ variance inflation factors (VIFs); those having a VIF score greater than 5 were excluded to avoid multicollinearity. Following this approach, we excluded two variables: access to basic water services (which covered 99% of households) and overcrowding.

**Table 1:** Variables included in the multiple logistic regression (MLR) models stratified by age (the models include individual-level observations from the two selected sites in Abidjan, Côte d'Ivoire)

Dependent variable	Stratification	Selected independent variables
Diarrhea: whether the individual had diarrhea in the 2 weeks preceding the survey (0 = no; 1 = yes)	MLR 1: general population: *N*_gen pop_ = 2043 individuals^1^ in the general population (two sites combined) MLR 2: under-5 stratum: *N*_under 5_ = 235 individuals^1^ in the population under 5 years (two sites combined)	• Exposure 1: individual lives in a dwelling with an indoor cooking space (0 = no; 1 = yes) • Exposure 2: individual lives in a dwelling built with inadequate materials (0 = no; 1 = yes) • Exposure 3: individual lives in a dwelling that had constant access to water in the month preceding the survey (0 = no; 1 = yes) • Control 1: individual lives in a dwelling with access to basic^2^ hygiene amenities (0 = no; 1 = yes) • Control 2: individual lives in a dwelling with access to basic^2^ sanitation amenities (0 = no; 1 = yes) • Control 3: head of household where individual lives has secondary education (0 = no; 1 = yes) • Control 4: individual lives in a relatively wealthy household (0 = no; 1 = yes)

^1^*N* corresponds to the number of individuals living in a household with valid answers for all 8 variables

^2^As defined by the WHO-UNICEF JMP for water, sanitation, and hygiene [27]

We assessed the spatial distribution of diarrheal cases and confronted it with the spatial distribution of deprived dwellings, based on the aforementioned housing variables. To do so, we detected clusters of households affected by diarrhea and each dwelling deprivation feature through local join count (LJC) statistics [30]. The latter consists of counting, for each point-observation where an event happens (e.g., diarrhea), the number of neighboring observations within a pre-defined search radius (“bandwidth”) where the same event happens. This count of events occurring in neighboring observations corresponds to the LJC. The definition of “bandwidth” depends on the spatial density of point observations and the analysis’s spatial resolution. As we analyzed small areas, we limited the bandwidth to the closest 16 observations, corresponding to a median distance between points considered “neighbors” of 36 m in Azito and 17 m in Williamsville. Finally, we ran an algorithm that randomly permutated the points’ positions to infer the probability of apparent clusters (i.e., observations with relatively high LJC values) being, in fact, due to chance (spatial randomness).

### Ethical Considerations

We obtained clearance from the Comité National d’Éthique des Sciences de la Vie et de la Santé in Côte d’Ivoire (ref. no. 005-22/MSHPCMU/CNESVS-km) and from the École Polytechnique Fédérale de Lausanne in Switzerland (decision no. 068-2020). Permission to use individual data was obtained through informed consent forms signed by the heads of households (adults only). In case they were illiterate, the information was read to them in front of a literate witness well-known to the participant.

## Results

We collected data from 567 households (266 in Azito and 301 in Williamsville), corresponding to 2498 individuals (1191 in Azito and 1307 in Williamsville). Among those individuals, 53% were female (*n* = 1328), and 11% (*n* = 283) were younger than 5 years. In Azito, the prevalence of diarrhea in the general population was 15% and 27% among children under 5 years old. In Williamsville, these prevalence rates were, respectively, 14% and 22%.

### Associations Between Diarrhea and Dwelling Deprivation Features

The MLRs revealed that, at constant access to basic hygiene and sanitation and constant socioeconomic levels, the selected dwelling deprivation features were consistently associated with diarrhea (Table 2). Overall, the aORs showed consistent trends for all variables, but most of the associations observed were only significant in the general population group (not meeting the significance criteria in the under-5 child stratum).

**Table 2:** Adjusted odds ratios (aOR) for cases of diarrhea reported in the two selected sites in Abidjan (February 2022), stratified by age group

Exposure	General population (*n* = 2043^1^)				Age < 5 years old (*n* = 235^1^)			
	aOR	Lower CI (95%)	Upper CI (95%)	Significance	aOR	Lower CI (95%)	Upper CI (95%)	Significance
Indoor cooking space	**0.55**	**0.42**	**0.73**	****^2^	0.70	0.35	1.42	Not significant
Inadequate dwelling materials	**1.79**	**1.31**	**2.44**	****^2^	2.06	0.98	4.33	*^2^
Water availability (month preceding survey)	**0.68**	**0.53**	**0.89**	***^2^	0.63	0.33	1.23	Not significant
Access to basic hygiene	**0.60**	**0.46**	**0.79**	****^2^	**0.37**	**0.18**	**0.73**	***^2^
Access to basic sanitation	1.20	0.89	1.62	Not significant	1.48	0.73	3.00	Not significant
Head of household with secondary education	**1.51**	**1.15**	**1.98**	***^2^	**2.11**	**1.09**	**4.08**	**^2^
Relatively wealthy household	**1.51**	**1.15**	**1.99**	***^2^	1.25	0.63	2.51	Not significant

Bold: statistically significant variables

^1^Corresponds to the number of individuals in the two sites living in a household with valid answers to all 8 variables (1 dependent variable and 7 control variables) included in the model

^2^The number of * indicates the significance of each beta coefficient resulting from the multiple logistic regression (MLR), which corresponds to the probability of the aOR being equal to 1: *P value < 0.1, **P value < 0.05, ***P value < 0.01, ****P value < 0.001

Living in a dwelling built with inadequate materials was consistently associated with higher odds of diarrhea (aORs > 1 in both groups), but the aOR only met the significance criteria in the general population group (aOR = 1.79, 95% CI: 1.31–2.44). Still, for children younger than 5 years, the lower 95% CI value was close to 1, showing a non-negligible, positive association with diarrhea. Cooking indoors was consistently associated with lower odds of diarrhea (aORs < 1 in both groups), but the aOR was only significant in the general population (aOR = 0.55, 95% CI: 0.42–0.73). Constant water availability in the month preceding the survey was also consistently associated with lower odds of diarrhea (aORs < 1 in both groups) but was only significant in the general population (aOR = 0.68, 95% CI: 0.53–0.89).

Regarding the control variables, most of them showed significant associations with diarrhea, notably access to basic hygiene amenities. Access to basic hygiene amenities was strongly associated with lower risks of diarrhea, both in the general population (aOR = 0.60, 95% CI: 0.46–0.79) and in children younger than 5 years (aOR = 0.37, 95% CI: 0.18–0.73). The household head’s education level was significantly associated with diarrhea, both in the general population (aOR = 1.51, 95% CI: 1.15–1.98) and in children younger than 5 years (aOR = 2.11, 95% CI: 1.09–4.08). The household’s asset-based wealth index was consistently associated with diarrhea (aORs > 1), but the aOR was only significant in the general population (aOR = 1.51, 95% CI: 1.15–1.99).

### Spatial Distributions of Diarrheal Cases and Dwelling Deprivation Characteristics

**Fig. 2:**
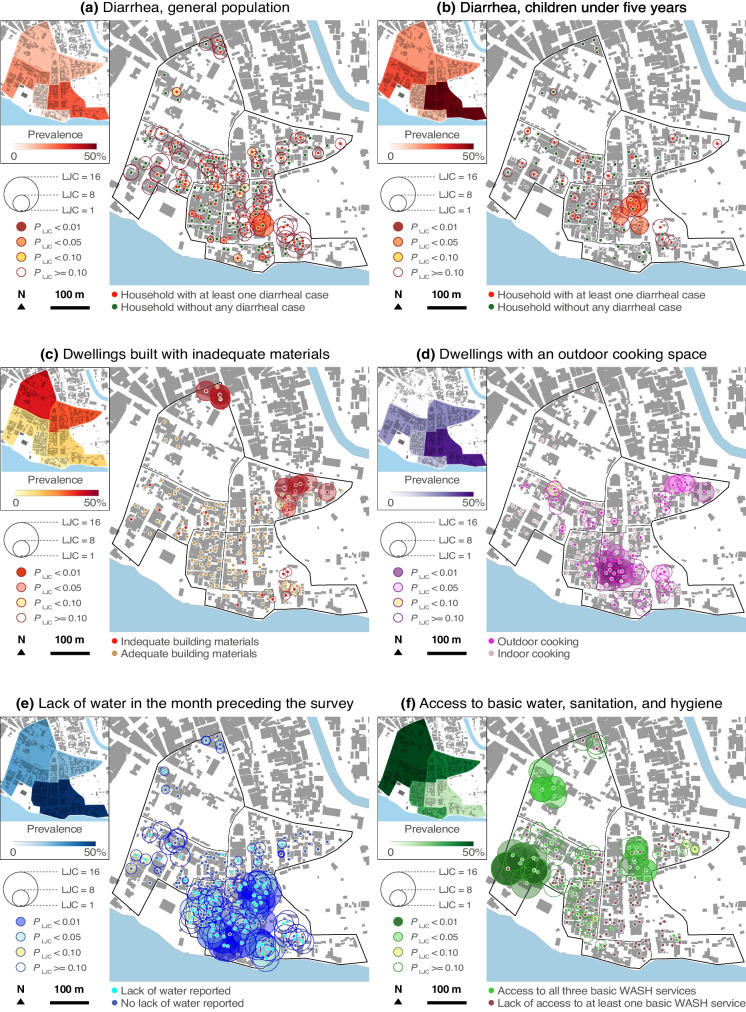
Spatial analysis in Azito (distribution of diarrheal cases and dwelling deprivation features in February 2022). Elaborated from Ecopia Building Footprints © 2021 Ecopia Tech Corporation, Imagery © 2021 DigitalGlobe, Inc

**Fig. 3:**
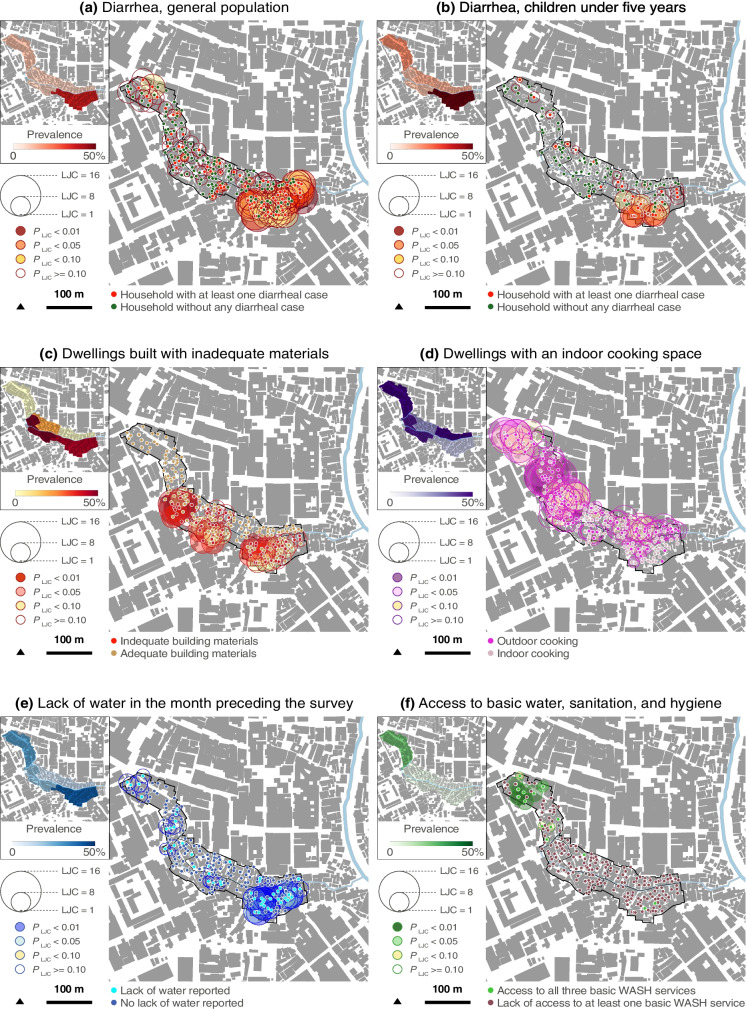
Spatial analysis in Williamsville (distribution of diarrheal cases and dwelling deprivation features in February 2022). Elaborated from Ecopia Building Footprints © 2021 Ecopia Tech Corporation, Imagery © 2021 DigitalGlobe, Inc

Although the two study sites occupy relatively small areas, we found significant within-site variations in the prevalence of diarrhea and the physical characteristics of the dwellings. Figures 2 and 3 depict these variations through LJC statistics characterized by the count number (circle size) and likelihood (circle color). Circles in darker colors indicate a statistically significant spatial concentration of an event (e.g., diarrhea case), that is, a cluster.

Overall, the spatial analysis corroborated the aORs. Sectors with more households affected by the lack of water, the lack of at least one basic WASH service, and the lack of an indoor cooking space corresponded to those more affected by diarrhea (both in the general population and in children younger than 5 years). Regarding the dwelling materials, the trends differed. In Williamsville, the location of inadequately built dwellings corresponded approximatively to that of diarrheal cases, while in Azito, their spatial distributions did not match.

In each site, we identified a sector that was more affected than others by dwelling deprivation and diarrhea. In Williamsville, the sector located in the southeast corner of the study site had significant clusters of diarrheal cases (both in the general population and children under 5 years), as well as significant clusters of households lacking access to basic WASH, constant water service, an indoor cooking space, and adequate building materials. In Azito also, the sector located in the southeast corner had significant clusters of diarrheal cases (both groups) and significant clusters of households lacking access to basic WASH, constant water service, and indoor cooking space.

## Discussion

### Dwelling Conditions and Diarrhea

Construction materials and the spatial disposition of cooking spaces were consistently associated with higher risks of diarrhea, even accounting for access to basic sanitation and hygiene amenities, continuity of water services, and socioeconomic status. These results corroborate previous findings regarding spatial predictors of diarrhea in Côte d’Ivoire [17], suggesting that housing conditions may play an important role in mediating contamination pathways that cause diarrhea.

Living in a dwelling built with inadequate materials may exacerbate exposure to environmental reservoirs of diarrheal pathogens, as indoor spaces are not effectively protected from external elements like wastewater or dirt [7, 31]. This observation is especially salient in contexts where basic sanitation coverage is limited and environmental exposure to pathogens is relatively high. Of relevance, although the association between diarrhea and inadequate dwelling materials showed consistent trends in the aORs, these events’ spatial distributions did not always coincide: they did in Williamsville but not in Azito. This observation might be explained by the different baseline levels of access to WASH services, which were much lower in Williamsville compared to Azito, making protection from environmental contamination sources more relevant in the former than in the latter.

Similarly to inadequate building materials, indoor cooking spaces might offer better protection from environmental contaminants by physically protecting food handling from external elements like flies or dirt. In fact, if food is exposed to such contamination sources, household members are exposed to higher risks of diarrhea independently of having access to basic hygiene facilities [10]. Moreover, protecting indoor spaces is critical when cohabitating with animals, as they pollute the environment with their feces, which in turn attract flies [9]. We observed that this was a common situation in Williamsville, notably in the areas having significant clusters of diarrheal cases. However, we note that indoor cooking with firewood or charcoal (very common in informal settlements) is negatively associated with respiratory health, especially in poorly ventilated spaces [32]. Therefore, the general health benefits of indoor cooking also depend on the type of fuel used, as well as the ventilation.

As for the continuity of water services (proxied by the constant availability of water during the month preceding the survey), it is a requirement to implement basic hygiene practices [27]. In fact, access to “basic” hygiene amenities (which include the presence of water and soap/detergent at the time of the survey) was consistently and significantly associated with lower risks of diarrhea across age groups. Having such amenities within premises surely facilitates handwashing, thus breaking contamination pathways at key moments (e.g., before eating and after defecation).

Surprisingly, access to basic sanitation had no significant association with diarrhea. This may be related to limitations in the data collected, which did not assess how excreta were managed. Another unexpected result was the significant association between heads of households with secondary education and higher risks of diarrhea. This may be due to a recall bias given by socioeconomic status, which has been detected elsewhere [28]. The same recall bias might explain the association we observed between asset-based wealth and diarrhea.

### Spatial Distribution of Housing Deprivation and Diarrhea

We located specific areas in each site that were disproportionately affected by diarrhea and dwelling deprivation. Such high-resolution information is crucial to guiding cost-effective interventions to improve housing and infrastructure and address intra-urban health inequities [33]. Moreover, we found that diarrheal cases followed spatial patterns very similar to those of dwelling deprivation features in the two study sites. This trend is visible in the prevalence and LJC maps (Figs. 2 and 3). The spatial coincidence observed between diarrhea and dwelling deprivation suggests that, even within small areas, differences in housing and habitat conditions might generate health inequities.

Given that the same settlement may have significant disparities within its boundaries regarding housing conditions and risk of diarrhea, it is essential to conceive of a spectrum of deprivation in the universe of informal settlements. Indeed, as previous studies besides this one have shown [16, 34], these communities are not homogenous and, hence, have different needs. These observations highlight the importance of having structures such as “urban health observatories” [35], for instance, the Nairobi Urban Health and Demographic Surveillance System (NUHDSS) in Kenya. These observatories collect high-resolution health data and, hence, are able to provide relevant intelligence to support efficient interventions.

### WASH Services Are Essential, But Not Enough: Policy Implications and Further Research

In informal settlements, the sole availability of “basic” WASH services might be insufficient to prevent diarrheal diseases. Our findings suggest that, although access to those services is essential, their potential health benefits may be hampered if housing conditions remain inadequate. Indoor spaces must be protected from external reservoirs of pathogens, especially in areas where people cohabitate with animals and with limited coverage of safely managed water and sanitation—i.e., safe management of excreta and constant delivery of potable water [27]. Against this background, “slum upgrading” projects should improve dwelling conditions in addition to upgrading urban infrastructure and services. As contamination pathways are multiple and vary across sites and populations [12, 31], only a combination of interventions may effectively prevent diarrheal diseases.

Furthermore, our results suggest that access to a “basic” water source does not guarantee continuous water service (i.e., availability whenever needed). Although 99% of the participating households had access to a “basic” water source, only 66% effectively had constant access to water during the month preceding the survey. This confirms the necessity of indicators simultaneously addressing the safety and availability of water in household questionnaires, as advised by the JMP [27] and as determined by Sustainable Development Goal 6, indicator 6.1.1 (proportion of population using safely managed drinking water services). Of relevance, we found that continuous access to water was consistently associated with lower risks of diarrhea in the two study sites. Given its public health implications, new research is needed to improve water distribution systems in the context of informal settlements.

### Study Limitations

Given our study’s cross-sectional design and the lack of a quantitative microbial risk assessment, our results cannot prove any causal relation between the selected housing deprivation variables and diarrhea. From an inferential standpoint, we acknowledge that the maps only show “circumstantial” associations between diarrhea and dwelling characteristics (events occurring in the same place simultaneously). Moreover, from an epidemiologic standpoint, we did not address the specific etiology of diarrheal cases and could not confirm them as they were self-reported. Although we did not have the resources to address the epidemiologic limitations, the inferential shortcomings were mitigated by combining the spatial analyses with the MLRs, which tested associations based on individual-level observations. This procedure did neither allow inferring causal associations nor confirming whether housing conditions mediate contamination pathways for diarrhea. However, our exploratory approach was helpful in identifying potential risk factors for diarrhea that have not been sufficiently examined from a spatial (cartographic) perspective.

## Conclusion

Our findings suggest that non-durable building materials, discontinuity of water service, and cooking outdoors constitute potential risk factors for diarrheal diseases in informal settlements. Although the construction and extension of safe WASH amenities are crucial to preventing diarrheal diseases, their health gains are limited if communities do not access dignified housing conditions. Planners should account for possible relations between contamination pathways causing diarrhea and the urban habitat by adopting an integrated approach that combines the upgrading of both WASH services and housing conditions.

We observed significant heterogeneity within and between the two study sites in Abidjan. Marked differences in housing characteristics coincided with different risk levels of diarrhea. These findings corroborate previous evidence pointing to the necessity of conceiving spatial determinants of the risk for diarrhea and, especially, of a “spectrum” of deprivation within the heterogeneous universe of informal settlements. The latter are, indeed, extremely diverse and require a setting-specific understanding to tailor interventions and maximize public health gains.

### Supplementary Information

Anonymized input data and Python notebooks are available at https://github.com/ceat-epfl/housing-deprivation-diarrhea.
